# Cocaine Induced Bilateral Posterior Inferior Cerebellar Artery and Hippocampal Infarction

**DOI:** 10.7759/cureus.2576

**Published:** 2018-05-04

**Authors:** Naresh Mullaguri, Anusha Battineni, Aarti Narayan, Raviteja Guddeti

**Affiliations:** 1 Neurology, Cleveland Clinic Foundation, Cleveland, USA; 2 Division of Pulmonary and Critical Care Medicine, Mayo Clinic, Rochester, MN, USA; 3 Cardiovascular Medicine, Creighton University School of Medicine

**Keywords:** hippocampal infarction, vasospasm, ischemic stroke, urine toxicology, cocaine related stroke, posterior inferior cerebellar artery infarction

## Abstract

Cocaine is one of the most commonly abused recreational drugs, second only to marijuana. It blocks the reuptake of neurotransmitters such as norepinephrine and dopamine, that leads to persistent post-synaptic stimulation responsible for its excitatory effects. Cocaine-related strokes, both ischemic and hemorrhagic, have been well described in the literature and cerebral vasospasm is hypothesized as one of the major mechanisms responsible for the presentation. Although cases of posterior circulation infarction were previously reported, we herein report a rare presentation of a cocaine-induced bilateral posterior inferior cerebellar artery and hippocampal infarction in a middle-aged female.

## Introduction

Cocaine is one of the most commonly abused recreational drugs with sympathomimetic activity. It is known to exert its effects by blocking the reuptake of neurotransmitters such as norepinephrine and dopamine, causing persistent post-synaptic stimulation responsible for its excitatory effects. Most common routes for abuse are nasal insufflation and smoking of its impure form called ‘crack cocaine’. Neurovascular complications of hemorrhagic and ischemic strokes related to cocaine abuse are well described in the literature. Cases of posterior circulation infarction were previously reported; however, cocaine-induced bilateral posterior inferior cerebellar artery and hippocampal infarction have not been reported to the best of our knowledge.

## Case presentation

A 66-year-old African-American female was brought to the emergency room (ER) for confusion. Her past medical history is significant for polysubstance abuse (heroin, prescription opioids) with multiple prior emergency room visits for heroin overdose, bacterial endocarditis 30 years ago with remote epidural abscess, cervical cord compression from C3-C6 and myelopathy with residual bilateral upper extremity contractures and lower extremity weakness, hepatitis C and chronic obstructive pulmonary disease. According to the patient’s daughter, she appeared somnolent a day prior to the admission. On the day of admission, she seemed confused with short-term memory loss, unable to recognize the daughter’s face along with significant receptive aphasia, although she was alert and conversing. She was unable to perform the usual activities of daily living. Due to concern for stroke, she was brought to the ER for evaluation. She denied a headache, fever, malaise, night sweats, and loss of weight lately. She denied any chest pain, palpitations, loss of consciousness or seizure-like activity.

**Figure 1:**
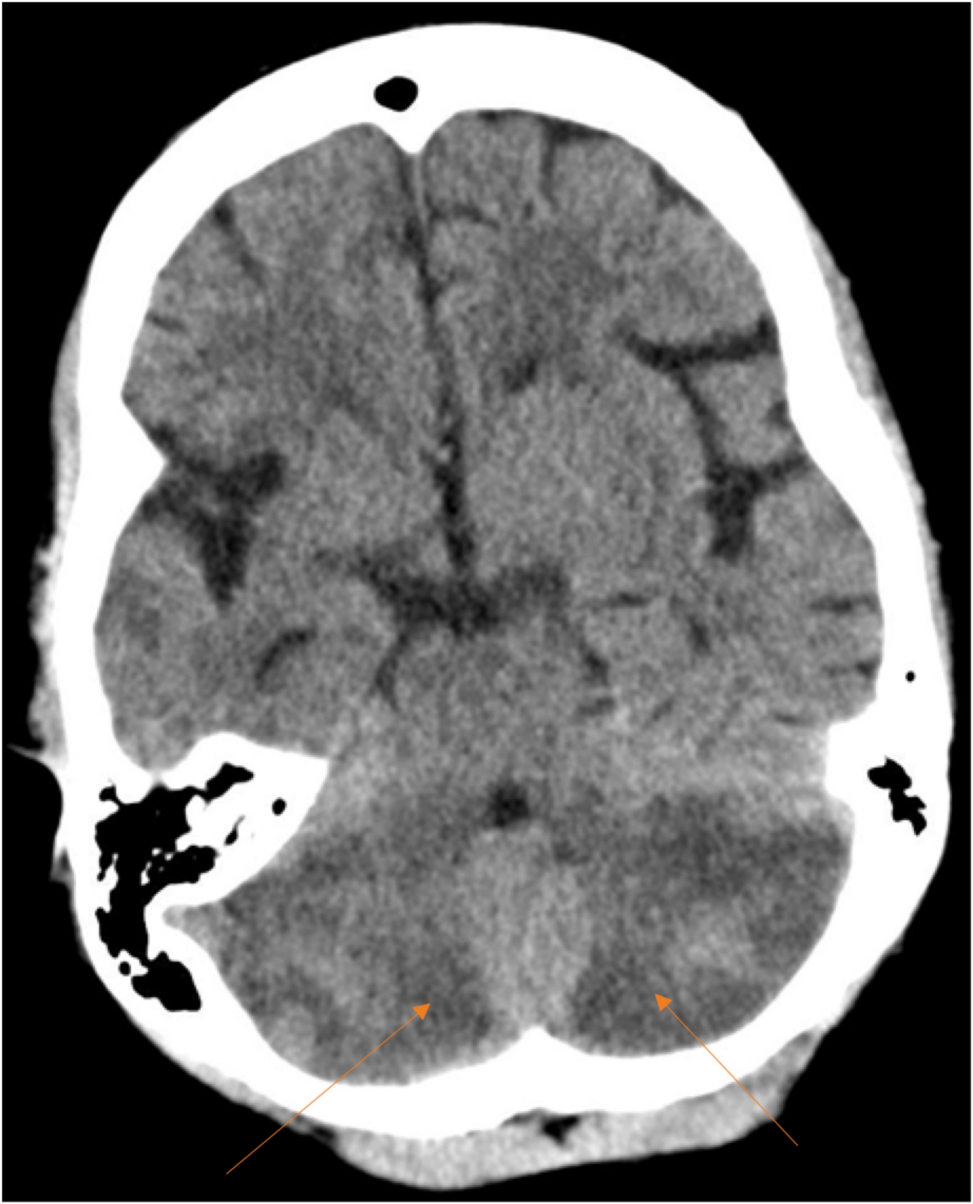
Computed tomography (CT) scan of the brain Computed tomography axial section showing bilateral posterior inferior cerebellar hemisphere hypodensity (arrows) suggestive of subacute infarction with no effacement of the fourth ventricle.

In the ER, she was afebrile with oxygen saturation of 100% on 4L of oxygen via nasal cannula, blood pressure was 157/96 mm Hg, heart rate of 92 beats per minute. Physical examination showed a middle-aged lady who was alert, oriented to name and place but not to time, along with mild receptive aphasia. Cranial nerves examination was unremarkable. Motor examination showed decreased bulk in bilateral upper extremities with moderate spasticity, tight contractures of the arms and forearms in flexed posture with some antigravity strength, bilateral lower extremity weakness with left side worse than right. Sensations were intact to light touch and pinprick in all the four extremities. Given the paucity of extremity strength, coordination and gait were difficult to assess. She scored 14 points on the National Institutes of Health Stroke scale assessment. Her initial laboratory work was significant for elevated liver transaminases (aspartate transaminase 1399 U/L (normal range 13-35 U/L) and alanine transaminase 894 U/L (normal range 7-38 U/L), elevated creatine kinase of 1790 U/L and was thought to be secondary to rhabdomyolysis. Serum magnesium and phosphorous were low at 1.5 mg/dL (normal range 1.7 -2.3 mg/dL) and 1.6 mg/dL (normal range 2.7-4.8 mg/dL) respectively. Urine toxicology was positive for cocaine. She had normal prothrombin time of 10.9 seconds (normal range 9.7-13.0 seconds), an international normalizing ratio (INR) of 1.1 (normal range 0.9-1.3) and activated partial thromboplastin time of 24.7 seconds (normal range 23.0-32.4 seconds). Computerized tomography (CT) of the brain showed heterogeneous low attenuation involving the entirety of bilateral cerebellar hemispheres with associated mass effect and partial effacement of the fourth ventricle suggestive of sub-acute infarction with areas of petechial hemorrhage along with encephalomalacia in the right insular and anterior operculum (Figure 1).

**Figure 2:**
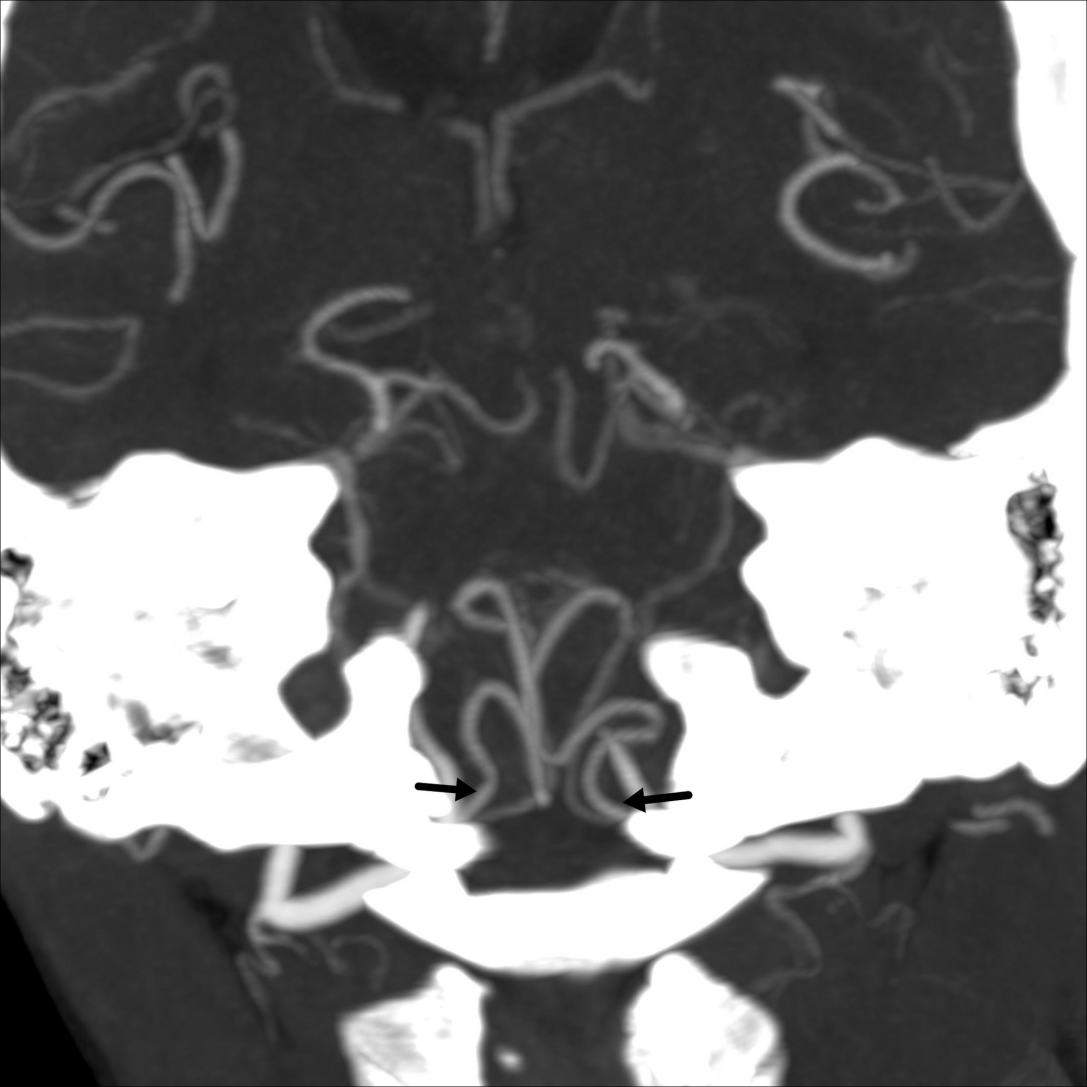
Computed tomography (CT) angiogram of the head Computed tomography angiogram of the head (maximal intensity projection images) coronal section showing patent bilateral posterior inferior cerebellar arteries arising from the intracranial vertebral arteries (black arrows).

There was no evidence of obstructive hydrocephalus. Due to the concern for posterior circulation large vessel occlusion, an emergent CT angiogram of the head and neck was performed which ruled out any dissection, significant stenosis or occlusion of intracranial or extracranial cerebral vasculature with areas of non-opacification of superior sagittal sinus and left transverse sinus. Both posterior inferior cerebellar arteries were widely patent (Figure 2).

**Figure 3:**
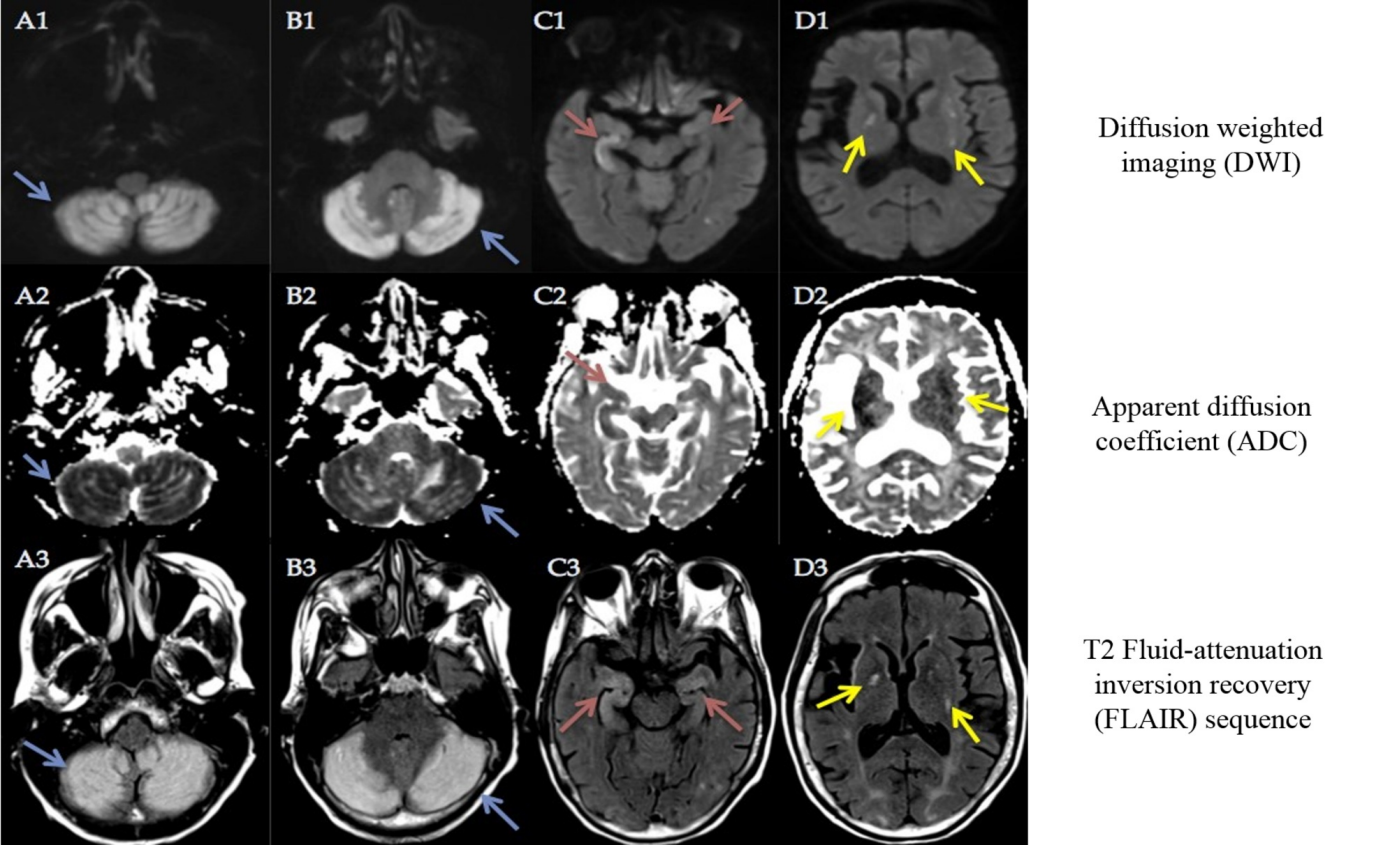
Magnetic resonance imaging (MRI) of the brain Magnetic resonance imaging of the brain DWI, ADC and T2 FLAIR sequences of the corresponding axial sections at cerebellar hemispheres (A1, A2, A3, B1, B2, B3), hippocampi (C1, C2, C3), and basal ganglia (D1, D2, D3) showing diffusion restriction and hyperintensity in the bilateral posterior inferior cerebellar artery (PICA) (blue arrows), hippocampi (red arrows), and basal ganglia regions (yellow arrows) respectively.

The differential diagnosis included multifocal infarction secondary to cocaine-related vasospasm, posterior reversible encephalopathy syndrome (PRES) and infective endocarditis with septic emboli (given prior history of heroin abuse). She was admitted to the neurointensive care unit for impending obstructive hydrocephalus and was started on hypertonic saline. Neurosurgery was consulted for possible external ventricular drain placement and posterior fossa decompression. She underwent magnetic resonance imaging (MRI) of the brain along with MR venogram which showed infarcts in the bilateral occipital lobes, both hippocampi and bilateral basal ganglia in addition to bilateral cerebellar hemispheres suspicious for an embolic phenomenon (Figure 3).

A magnetic resonance venogram ruled out dural venous sinus thrombosis. Cerebral angiography was deferred given normal CT angiography. Transthoracic echocardiogram showed ejection fraction of 59% with no regional wall motion abnormalities. Two sets of blood cultures drawn on the day of admission showed no growth. Her ultra-sensitive C-reactive protein (CRP) was elevated at 10.4 mg/L (reference range <3.1 mg/L) but erythrocyte sedimentation rate (ESR) was normal at 2 mm/hr (reference range 0-20 mm/hr). Telemetry during the course of hospitalization was unremarkable for arrhythmias. Her glycated hemoglobin (HbA1c) was 5.4% (normal range 4.3%-5.6%), low-density lipoprotein (LDL) was 105 mg/dL (normal range 60-129 mg/dL). Acute hepatitis viral panel showed positive hepatitis C virus (HCV) antibody with elevated Hepatitis C virus ribonucleic acid (HCV RNA) by polymerase chain reaction (PCR) 4,838,806 IU/mL. She was diagnosed with cocaine-induced bilateral posterior inferior cerebellar artery and hippocampal infarction with multifocal punctate infarcts in the bilateral posterior cerebral artery and basal ganglia secondary to severe vasoconstriction. PRES was a consideration given the significant involvement of posterior circulation and mild encephalopathy, but she did not have high blood pressure during hospitalization, denied headache, vision issues and no pattern of radiographic vasogenic edema, but rather multifocal infarcts causing mild aphasia and encephalopathy. Due to negative blood cultures, normal echocardiogram and lack of fever and other constitutional symptoms, we ruled out infective endocarditis.

Her mental status improved during hospitalization and she was discharged to a skilled nursing facility after seven days with persisting memory problems and unable to recognize faces. Her modified Rankin scale at the time of discharge was 4, requiring assistance with most of her activities of daily living and unable to walk. She was lost to follow up.

## Discussion

Cocaine is the second most commonly abused recreational drug after cannabis [1]. The overall prevalence of cocaine use among US adults is 1.5%. It is a well-recognized cause of ischemic brain damage, especially in the young to middle-aged adults. The use of cocaine, although has declined over the years, has been associated with a myriad of pathologies ranging from an acute coronary syndrome, ischemic stroke, heart failure, arrhythmias and aortic dissection. Within the last two decades, the incidence of cocaine-related strokes has increased by 19% [2]. A recent National Inpatient Sample study by Desai et al. on the impact of cocaine use on acute ischemic stroke patients noted cocaine use to be a major risk factor for hospital admission for acute ischemic stroke [3]. In this study, cocaine use was associated with higher in-hospital mortality. Interestingly, this study noted that the acute ischemic stroke with cocaine group consisted of a higher number of older patients aging >85 yrs old. The authors speculate that this could be secondary to the cumulative effect of traditional risk factors and long-term effects of cocaine. Previously, several case reports have been reported on the association of cocaine with hippocampal, basilar artery, basal ganglia and cerebellar strokes (Table 1) [4-12].

**Table 1:** Reported cases of cocaine use-related posterior circulation stroke TTE: transthoracic echocardiogram; CUS: carotid ultrasound; MRA: magnetic resonance angiography; MDMA: methylenedioxymethamphetamine; RCVS: reversible cerebral vasoconstriction syndrome.

Author	Age/Sex	Stimulant	Symptoms	Neuroimaging	Cardiac Imaging	Mechanism
Vidal et al. [4]	--	Cocaine	Disorientation	Bilateral hippocampi, left basal ganglia, left inferior cerebellum		Possible vasospasm
Aggarwal et al. [5]	25/Female	Cocaine	Unsteady gait, headache, dizziness, vomiting	Normal cerebral angiogram, left inferior cerebellar infarction on MRI		Possible vasospasm
Connelly et al. [6]	44/Male	Cocaine	Confusion, memory loss	Bilateral hippocampi and centrum semiovale, CUS normal	TTE normal	Hypoxia
Vallee et al. [7]	25/Female	Cocaine, ecstasy	Confusion, dysarthria, right hemiplegia, coma	Mid to distal basilar artery occlusion s/p thrombectomy		Thrombosis
Alqahtani et al. [8]	75/Male	Cocaine	Coma, extensor posturing of right upper extremity	Bilateral pontine infarct, basilar artery thrombus	TTE normal	Possible vasospasm
Surpur et al. [9]	36/Male	Cocaine	Headache	4 vessel extracranial dissection with diffuse vasospasm, RCVS		Vasospasm, dissection
Bolouri et al. [10]	25/Female	Cocaine	Unresponsiveness, asystole	Hippocampi, Globus pallidi, MRA normal	TTE normal	Possible vasospasm
Daras et al. [11]	21/Male	Cocaine	Dysarthria, quadriparesis, cranial nerve palsies (bilateral 7th, left 3rd, right 6th)	Midbrain infarction, normal angiogram	TTE normal	-
	36/Male	Cocaine	Ataxia	Right cerebellum and left occipital lobe infarct		-
	29/Male	Cocaine	Aphasia, right hemiparesis	Hemorrhagic infarct, left basal ganglia		-
Haut et al. [12]	55/Male	Cocaine, Methamphetamine, MDMA	Unresponsiveness, impaired explicit and procedural memory	Bilateral hippocampal and basal ganglia infarction		Vasospasm

However, bilateral involvement of the cerebellum and hippocampus have not been reported in the literature so far. In our patient, we noted diffusion restriction of both cerebellar hemispheres and hippocampi on MRI brain. 

Cocaine principally exerts its activity by inhibiting norepinephrine, dopamine and 5-hydroxytryptamine reuptake. This leads to heightened sympathomimetic activity causing tachycardia, arrhythmias, acute coronary syndrome, and even sudden death. Several mechanisms for cocaine-related ischemic stroke have been proposed among which transient cerebral vasospasm, vasculitis, cardioembolic phenomena from infective endocarditis and arrhythmias constitute the majority [3,13]. Cocaine crosses blood-brain barrier faster than other stimulant drugs. Cerebral vasospasm is known to affect both larger cranial arteries and cortical microvasculature. Animal studies have demonstrated complete vascular occlusion on cerebral angiography from the intense vasoconstriction effects of cocaine leading to global or focal ischemia [14]. Reduction in cerebral metabolism from chronic cocaine use may lead to downregulation of cerebral blood flow. The ischemic injury to the hippocampal neurons is thought to be secondary to the selective vulnerability of CA1 neurons to hypoxia along with dopaminergic excitotoxicity [15]. Other potential mechanisms include vascular thrombosis and hypoxia from respiratory arrest. Cerebral vascular thrombosis can also result from vasospasm secondary to endothelial injury and platelet aggregation. Although poorly understood, vasculitis-related chronic cerebrovascular changes possibly including moyamoya, which can lower the threshold for ischemic stroke, have also been reported in the literature [16]. Also, cocaine, when consumed in association with alcohol, is known to have prolonged euphoric effects due to the production of cocaethylene, which has a longer half-life than cocaine.

Imaging characteristics of ischemic stroke related to cocaine use are similar to other causes of stroke. In our patient, CT angiogram of the head showed patent posterior inferior cerebellar arteries suggesting possible vasospasm as the potential cause of the patient’s bilateral cerebellar infarction. Although our patient’s MRI findings of infarcts in the occipital lobes, hippocampi, basal ganglia and cerebellar hemispheres suggest possible embolic source but also known to occur with vasospasm of cerebral blood vessels. Moreover, cardiac workup showed no evidence of intramural thrombus, endocarditis or atrial fibrillation in our patient. Prior published case reports of cocaine-related posterior circulation infarction in the inferior cerebellum, hippocampi and basal ganglia are proposed to be secondary to vasospasm as in most of these patients, the angiography showed patent vessels [5,11]. Johnson et al., using novel quantitative single photon emission CT method for measuring brain blood flow, demonstrated that intravenous cocaine use is associated with a global reduction in brain blood flow. However, the reduction in blood flow was more pronounced in the dopamine-rich regions of the brain such as prefrontal, frontal, temporal and subcortical gray matter [17]. We hypothesize that posterior inferior cerebellar artery, posterior cerebral arteries, and lenticulostriate arteries are selectively prone to transient vasospasm due to the high adrenergic state from increased circulating catecholamines affecting their autoregulation. Hippocampi are involved in other disorders such as transient global amnesia and one of the proposed mechanism is due to increased sympathetic activity causing anterograde amnesia and neuroimaging evidence of punctate hippocampal lesions [18]. Cocaine might affect the hippocampi in a similar way by causing transient high catecholaminergic state. Posterior circulation is also selectively affected by disorders of cerebral autoregulation failure such as RCVS and PRES [19-20]. The proposed mechanism in both these disorders is uncontrollably high blood pressure, which is known to occur with cocaine intoxication.

Cerebellar strokes are associated with increased morbidity and mortality when compared with other forms of ischemic stroke most commonly resulting from hydrocephalus and compression of the adjacent brain stem. Fortunately, our patient did not develop obstructive hydrocephalus or tonsillar herniation, which usually entails the use of hyperosmolar therapy, and surgical intervention, which was a feared complication of posterior inferior cerebellar artery infarction.

## Conclusions

Cocaine abuse is a major cause of acute ischemic stroke across all ages. Bilateral posterior inferior cerebellar artery, hippocampi, and basal ganglia infarction are rare but can occur in patients abusing cocaine possibly secondary to vasospasm and warrants toxicology screening.
